# Exercise Training Reduces Cardiac Dysfunction and Remodeling in Ovariectomized Rats Submitted to Myocardial Infarction

**DOI:** 10.1371/journal.pone.0115970

**Published:** 2014-12-31

**Authors:** Simone Alves de Almeida, Erick Roberto Gonçalves Claudio, Vinícius Franskoviaky Mengal, Suelen Guedes de Oliveira, Eduardo Merlo, Priscila Lang Podratz, Sônia Alves Gouvêa, Jones Bernardes Graceli, Gláucia Rodrigues de Abreu

**Affiliations:** 1 Departamento de Ciências Fisiológicas, Centro de Ciências da Saúde, Universidade Federal de Espírito Santo, Vitória-ES, Brasil; 2 Departamento de Morfologia, Centro de Ciências da Saúde, Universidade Federal do Espírito Santo, Vitória-ES, Brasil; Texas A&M University Health Science Center, United States of America

## Abstract

The aim of this study was to evaluate whether exercise training (ET) prevents or minimizes cardiac dysfunction and pathological ventricular remodeling in ovariectomized rats subjected to myocardial infarction (MI) and to examine the possible mechanisms involved in this process. Ovariectomized Wistar rats were subjected to either MI or fictitious surgery (Sham) and randomly divided into the following groups: Control, OVX+SHAM_SED_, OVX+SHAM_ET_, OVX+MI_SED_ and OVX+MI_ET_. ET was performed on a motorized treadmill (5x/wk, 60 min/day, 8 weeks). Cardiac function was assessed by ventricular catheterization and Dihydroethidium fluorescence (DHE) was evaluated to analyze cardiac oxidative stress. Histological analyses were made to assess collagen deposition, myocyte hypertrophy and infarct size. Western Blotting was performed to analyze the protein expression of catalase and SOD-2, as well as Gp91phox and AT1 receptor (AT1R). MI-trained rats had significantly increased in +dP/dt and decreased left ventricular end-diastolic pressure compared with MI-sedentary rats. Moreover, oxidative stress and collagen deposition was reduced, as was myocyte hypertrophy. These effects occurred in parallel with a reduction in both AT1R and Gp91phox expression and an increase in catalase expression. SOD-2 expression was not altered. These results indicate that ET improves the functional cardiac parameters associated with attenuation of cardiac remodeling in ovariectomized rats subjected to MI. The mechanism seems to be related to a reduction in the expression of both the AT1 receptor and Gp91phox as well as an increase in the antioxidant enzyme catalase, which contributes to a reduction in oxidative stress. Therefore, ET may be an important therapeutic target for the prevention of heart failure in postmenopausal women affected by MI.

## Introduction

Clinical and experimental studies have demonstrated that ovarian hormone deficiency results in an increased risk of cardiovascular disease (CVD). [1], [2] Coronary artery diseases, including acute myocardial infarction (MI), are an important cause of both mortality and disability in women, primarily those in the post-menopausal period, a period characterized by a fall in ovarian hormones production. [3]

The ventricular remodeling process after MI seems to occur differently in women because of the presence of ovarian hormones, primarily 17β-estradiol. [4] Experimental studies have shown that the absence of these hormones after MI is directly related to a worsening of autonomic dysfunction, [5] an increased time of contraction and relaxation of the right ventricle [6], an increased aortic reactivity to phenylephrine and a reduction in nitric oxide (NO) bioavailability. [7] Moreover, studies of women in the menopausal and postmenopausal periods showed reductions in systolic function and ejection fraction and an increase in the apoptotic cascade after MI, [4] all of which contribute to a worse prognosis for women affected by MI during this period.

Among the main factors that contribute to remodeling after MI or ovariectomy (OVX), the renin angiotensin system (RAS) seems to play an essential role, acting on collagen synthesis and degradation via activation of the AT1 receptor of angiotensin II (AngII), [8] as well as increasing reactive oxygen species (ROS) production, creating an oxidative stress environment. [9], [10], [11] After MI, an increase in oxidative stress biomarkers in both infarcted and non-infarcted areas suggests that ROS play an important role in many steps of the remodeling process after MI, including an exacerbation of the inflammatory response, as well as hypertrophy and apoptosis of cardiomyocytes. [12]

Physical exercise has become a non-pharmacological therapeutic option in the treatment of CVD and has been recognized as a relevant strategy for the prevention and reduction of pathological remodeling after MI. [13], [14] In patients with stable heart failure subjected to a physical training routine, an improvement in symptoms and an increase in exercise tolerance were observed, as well as a positive impact on quality of life and a decrease in the number of hospitalizations. [15] Beneficial effects were seen in experimental studies with MI induction, including a reduction in ventricular hypertrophy and a restoration of contractility, [16] as well as a reduction in mitochondrial dysfunction, [17] an increase in antioxidant enzyme activity, [18] an increase in parasympathetic activity, [5] and a decrease in circulating levels of Ang II. [19]

Nevertheless, the majority of experimental studies that have assessed the effects of physical exercise after MI were performed either in male animals or in females with intact ovaries; therefore, it was not possible to assess the effects of physical training on cardiac function after MI in the absence of ovarian hormones.

The aim of this study was to determine if exercise training prevents or minimizes cardiac dysfunction and pathological ventricular remodeling in ovariectomized rats subjected to MI. Moreover, we analyze a possible mechanism that may be associated with such effects.

## Methods

### Animals

Female normotensive Wistar rats of 8 weeks of age and weighing between 200–250 g were provided by university facility. The animals were kept in cages with free access to both water and standard rat chow (Purina Labina, SP, Brazil), under controlled temperature (22–24°C), humidity (40–60%) and light-dark cycle (12–12 h) conditions. Experiments were conducted in accordance with the Guide for the Care and Use of Laboratory Animals published by the US National Institutes of Health (NIH Publication, revised 1996), and efforts were made to minimize the animals' suffering. All procedures were approved by the Institutional Ethical Committee for Animal Care and Use of the Federal University of Espírito Santo under protocol number 059/2012. At the time of myocardial infarction surgery, the animals were randomly divided into one of the following groups (n = 12): control (CON); ovariectomized and sham infarct (OVX+SHAM_SED_); ovariectomized, sham infarct and exercise training (OVX+SHAM_ET_); ovariectomized and infarct (OVX+MI_SED_) and ovariectomized, infarct and exercise training (OVX+MI_ET_).

### Ovariectomy

Ovariectomy was performed under general anesthesia with a mixture of ketamine (50 mg/kg) and xylazine (10 mg/kg) *i.p.* A bilateral dorsolateral incision was made through skin, and the underlying muscle was dissected to locate the ovaries and fallopian tubes. The tubes were ligated with a suture line, and the ovaries were removed. The muscle and skin were then sutured with an absorbable suture. After the surgery, animals received an injection of antibiotics (2.5% enrofloxacin, 10 mg/Kg) *i.m.* In control group, a fictitious surgery was performed. All animals were taken for surgery in the same time period.

### Myocardial Infarction Procedure

One week after ovariectomy, rats were anesthetized with a mixture of ketamine (50 mg/kg) and xylazine (10 mg/kg) *i.p*, and under fully anaesthetized conditions (confirmed by the absence of corneal reflex), MI was produced as previously described by Pfeffer et al. [20] Briefly, a left thoracotomy was performed at the fourth left intercostal space, and the heart was quickly exposed. The left coronary artery was permanently occluded with a mononylon suture and the heart was then returned to its initial position, and the thorax was closed. Sham-operated (Sham) animals were subjected to all procedures, except coronary artery occlusion. The control group also underwent fictitious surgery for infarction. Seventy three percent of the rats who underwent surgery survived after completion of the protocol. Two MI rats and two MI+ET rats died before the end of the protocol.

### Exercise Training Protocol

Exercise training was performed on a motorized treadmill (EP 131, Insight, Brasil). The training protocol consisted of a modification of a protocol used previously for training MI rats. [5] Two weeks after infarction, the animals subjected to exercise training were adapted to a treadmill for one week (10 min/d; 0.3 Km/h); the regimen increased daily by ten minutes until reach sixty minutes on the fifth day. From the second week on, exercise duration was constant (60 min/day). The intensity was gradually increased in speed from 0.3 to 1.2 km/h, and performed 5 times per week, with two days of rest during the 8 week period. Animals rested for 48 h (to analyze the effects of chronic exercise) before undergoing hemodynamic evaluation.

### Measurement of Cardiac Function

After the final of training period, the animals were anesthetized with ketamine (50 mg/kg) and xylazine (10 mg/kg) for left ventricle catheterization. Briefly, the right common carotid artery was separated from connective tissue and catheterized with a fluid-filled polyethylene catheter (PE50). The catheter was connected to a pressure transducer (FE221 Bridge amp, ADInstruments, Australia) and a digital system (Powerlab 4/35, ADInstruments, Australia). After arterial systolic and diastolic blood pressures were recorded, the catheter was advanced into the left ventricle to obtain the following measurements: heart rate (HR), left ventricular systolic pressure (LVSP), end-diastolic pressure (LVEDP), and the maximum rate of pressure rise (+dP/dt) and fall (-dP/dt). It was not possible to measure other parameters related to cardiac function such as cardiac output and ejection fraction because we not evaluate the ventricular volume. However, other studies have been demonstrated that LVEDP presents as an important parameter for the assessment of ventricular function, and his increase is associated with ventricular dysfunction. [21] The heart, soleus muscle, abdominal fat, uterus and a lung were removed immediately after hemodynamic evaluation and weighed.

### Detection of superoxide production (Dihydroethidium fluorescence)

Unfixed frozen sections from the heart (n = 4 per group) were cut into 8-µm-thick sections and mounted on gelatin coated glass slides. Samples were incubated with the oxidative fluorescent dye dihydroethidium (DHE, 2 µmol/L) in a modified Krebs's solution (containing 20 mM HEPES), in a light-protected humidified chamber at 37°C for 30 min, to detect superoxide. The intensity of fluorescence was detected at 585 nm and quantified in the tissue sections using a confocal fluorescent microscope (Leica DM 2500 TI, Nikon Instruments Inc., Melville, NY, USA) by an investigator blinded to the experimental protocol. Analysis of 15 fields per sample were performed.

### Western Blotting Analyses

The hearts were homogenized in lysis buffer containing (mmol/l) 150 NaCl, 50 Tris-HCl, 5 EDTA.2Na, and 1 MgCl_2_ plus protease inhibitor (Sigma Fast; Sigma, USA). The protein concentration was determined by the Lowry method, [22] and bovine serum albumin (BSA) was used as the standard. Equal amounts of protein (50 µg) were separated by 10% SDS-PAGE. Proteins were transferred to polyvinylidene difluoride membranes incubated with mouse monoclonal antibodies for catalase (CAT; 1∶2000; Sigma, USA), rabbit polyclonal antibodies for superoxide dismutase (SOD-2; 1∶1000; Sigma, USA) and Gp91phox (1∶1000; BD, New Jersey, EUA) and rabbit polyclonal antibodies for AT_1_ (1∶500; Santa Cruz Biotechnology, CA, USA) and GAPDH (1∶1000; Santa Cruz Biotechnology, CA, USA). After washing, the membranes were incubated with either an alkaline phosphatase conjugated anti-mouse IgG (1∶3000, Abcam Inc., Cambridge, MA, USA) or an anti-rabbit antibody (1∶7000; Santa Cruz Biotechnology, CA, USA). The bands were visualized using a NBT/BCIP system (Invitrogen Corporation, CA, USA) and quantified using *ImageJ* software (National Institute of Health, NIH). The results were calculated using the ratio of the density of specific proteins to the corresponding GAPDH.

### Determination of Myocyte Hypertrophy and Fibrosis

After hemodynamic recordings, the heart was removed and rapidly washed with cold saline solution, and the ventricles were separated from the atria, blotted dry and weighed. The left ventricle was divided into three slices of approximately 2 mm, slices that were subsequently prepared for histology. Each slice was serially cut into 4-µm-thick transverse sections and stained with Sirius red to determine its collagen volume fraction (CVF). Slices were also stained with hematoxylin-eosin (H&E) to determine myocyte cross sectional area (MCSA). The percentage of Picrosirius red staining, which indicated CVF, was measured in images obtained with a digital camera (Evolution, Media Cybernetics, Inc., Bethesda, MD) coupled to an optical microscope (Eclipse 400, Nikon) under 400× magnification. Nine areas of high-power fields were analyzed in the subendocardial layer, and nine were analyzed in the subepicardial layer. For MCSA evaluation, 40 to 60 myocytes positioned perpendicularly to the plane of the section and having both a visible nucleus and a clearly outlined and unbroken cell membrane were selected in each animal. Cell images viewed with a video camera were projected onto a monitor and traced. Images for CVF and MCSA evaluation were processed with *ImageJ* software (v. 1.43u, National Institutes of Health, USA). Sections stained with Picrosirius were used to obtain 50 photomicrographs from heart tissue with a 40x objective lens. The areas were randomly selected, although fields containing medium-sized blood vessels were carefully avoided. [14], [23]

### Myocardial Infarction Extension

To analyze myocardial infarction extension, the LV was divided into transverse sections and stained with Picrosirius. After this procedure, the image sections were scanned (Laserjet Pro M1132, HP, USA) and analyzed with Image J software (National Institutes of Health, USA). The epicardial perimeter related to the infarcted area (delimited by Picrosirius coloring) was delimited. All of the procedures were repeated for the endocardium. Infarction extension was presented as the mean percentual value of the infarcted perimeter of the LV.

### Citrate Synthase Activity

Soleus muscles were homogenized in phosphate buffer (50 mM sodium phosphate, 1 mM EDTA and protease inhibitor cocktail (Sigma-Aldrich, USA), pH 7.4) and centrifuged for 15 minutes at 12000 g and 4°C; the pellet was then discarded, and the supernatant was used for the assay. The assay mixture contained 100 mM Tris, 1 mM EDTA, 0.2 mM DTNB, 0.1 mM acetil-CoA, 1% (v/v) Triton X-100, sample (130 mg of soluble proteins per mL of total assay) and 0.5 mM oxaloacetate (added last), as originally described. [24] Sample absorbance was monitored at 412 nm in a 96-well plate for 10 minutes at 25°C, and maximal citrate synthase activity was measured within the linear range of the assay.

### Statistical Analysis

Data are reported as mean ± SEM. Data for organ weights, protein expression and hemodynamic parameters were analyzed by one-way analysis of variance (ANOVA), and training was considered the main factor, followed by Fisher's post-hoc test for multiple comparisons. Non parametric tests were used to analyze histological data. Mann-Whitney test was used to compare infarct size. *P*<0.05 was considered statistically significant.

## Results

### Surgery and Training Efficacy

Uterus weight (UW) was used to determine estrogenic status. As expected, there was a significant decrease in all of these parameters in OVX animals compared with the control group. The effectiveness of exercise training was demonstrated by increased citrate synthase activity in the trained groups (Table 1).

**Table 1:** Morphometric parameters eight weeks after myocardial infarction and exercise training in ovariectomized rats.

	Control	OVX+SHAM_SED_	OVX+SHAM_ET_	OVX+MI_SED_	OVX+MI_ET_
	(n = 12)	(n = 12)	(n = 12)	(n = 12)	(n = 12)
BW initial (g)	232±5,74	233±11,29	250±8,84	240±10,96	228±7,38
BW final (g)	268±8,92	333±6,06 #	312±6,52 #	324±8,13 #	304±15,47 # ‡
BW range (%)	15±0,49	33±2,08 # †	20±2,00	26±2,38 #	30±2,31 # †
Fat abdominal deposits (g)	16,7±1,67	20,2±2,20	19,0±1,23	25,0±1,68 # †	17,2±1,85 $
UW (mg)	0,521±0,02	0,121±0,008 #	0,098±0,005 #	0,090±0,003 #	0,090±0,003 #
RVW Dry (g)	0,03±0,002	0,04±0,001	0,03±0,002	0,05±0,008 # ‡	0,05±0,003 # ‡
lung (g)	1,48±0,07	1,70±0,05	1,65±0,08	3,07±0,27 # ‡ †	2,75±0,26 # ‡ †
lung/BW (mg/g)	5,46±0,28	5,05±0,23	5,31±0,21	8,95±0,71 # ‡ †	7,15±0,66 # ‡ † $
% lung water	79,9±0,29	79,7±0,31	79,4±0,41	81,9±0,48 # ‡ †	81,9±0,45 # ‡ †
CS activity (µmol.mg^−1^.min^−1^)	3270±242	4105±533	5870±583# ‡	2933±457†	4753±273# $

Data are expressed as mean±SEM. BW, body weight; UW, uterine weight; RV, right ventricle; CS, Citrate Synthase.

# *P*<0.05 vs Control;

† *P*<0.05 vs OVX+SHAM_ET_;

‡ *P*<0.05 vs OVX+SHAM_SED_;

$ *P*<0.05 vs OVX+MI_SED_.

### Body Weight and Adiposity

Body weight was similar between the experimental groups at the beginning of the protocol. At the end of protocol, the OVX+SHAM_SED_, OVX+SHAM_ET_, OVX+MI_SED_ and OVX+MI_ET_ groups presented with increased body weight compared with the control group. However, OVX+MI_ET_ presented with reduced body weight compared with OVX+SHAM_SED_. Furthermore, analysis of total fat deposits showed an increase in the OVX+MI_SED_ group compared with the Control and OVX+SHAM_ET_ groups, although these deposits were significantly reduced in OVX+MI_ET_ compared with OVX+MI_SED_, demonstrating the efficacy of ET in reducing adiposity even after MI (Table 1).

### Myocardial Infarction Extension

**Figure 1:**
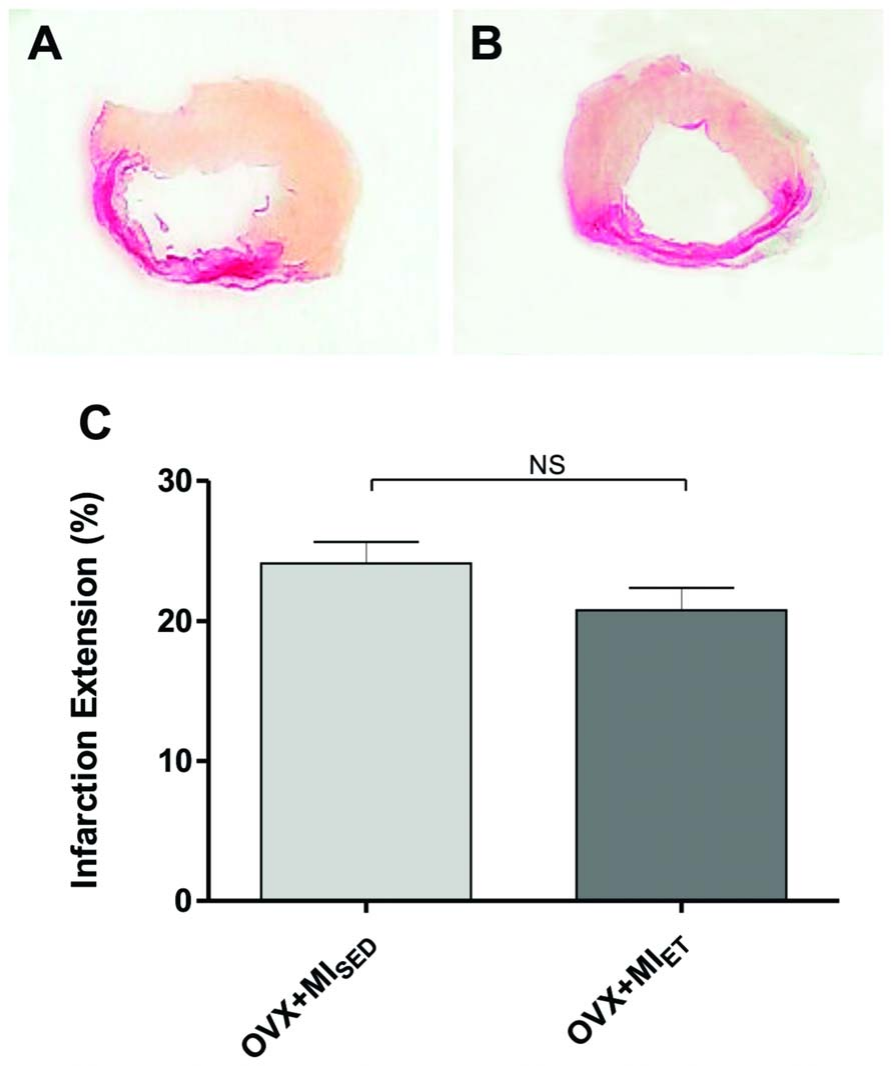
Histological analysis of myocardial infarction area. Infarcted area analysis showing no differences among groups. Data are expressed as mean ± SEM (n = 7). *P<0.05.

Ten weeks after coronary occlusion, infarction extension was assessed by staining transverse sections of the LV with Picrosirius. No animals of the Control, OVX+SHAM_SED_ and OVX+SHAM_ET_ group showed any visible area stained by Picrosirius. Fig. 1 showed representative histological sections of infarction extension.

### Cardiac Function

**Figure 2:**
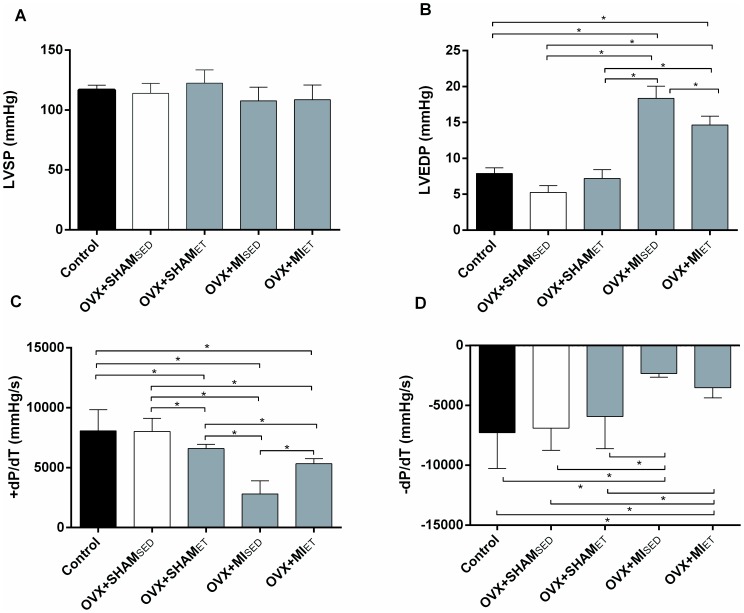
Cardiac function measurements. (A) Left ventricular systolic pressure (LVSP) demonstrating no differences among groups. (B) Exercise training reduced left ventricular end diastolic pressure (LVEDP) in OVX+MI_ET_ group compared to MI which occurs in parallel with an increased +dP/dt (C). There were a decrease in -dP/dt in the MI group compared with other groups which was not restored by ET (D). Data are expressed as mean ± SEM (n  =  12). *P<0.05.

There was a reduction in the heart rate of the OVX+SHAM_ET_ group compared to the OVX+SHAM_SED_ and Control groups, an expected adaptive response to ET. However, ET didn't prevent an increase in the heart rate of the OVX+MI_ET_ group compared to the SHAM+ET group (Table 2). The LVEDP increased significantly in the infarcted groups, in conjunction with reduced peak +dP/dt and -dP/dt (Fig. 2B, C and D respectively). These data confirm the progression of heart failure. However, the increase in LVEDP and the reduction in +dP/dt was attenuated in animals subjected to 8 weeks of exercise training (Fig. 2B and C respectively).

**Table 2:** Hemodynamic parameters eight weeks after myocardial infarction and exercise training in ovariectomized rats.

	Control	OVX+SHAM_SED_	OVX+SHAM_ET_	OVX+MI_SED_	OVX+MI_ET_
	(n = 12)	(n = 12)	(n = 12)	(n = 12)	(n = 12)
HR (bpm)	202±6	193±9	167±9 # ‡	200±9 †	213±6 †
SBP (mmHg)	117±6	135±6	129±5	107±6 ‡ †	129±3 $
DBP (mmHg)	83±5	106±5 #	104±4 #	85±4 ‡ †	99±3 # $
MAP (mmHg)	93±4	114±6 #	115±4 #	93±6 ‡ †	112±3 # $

Data are reposted as mean±SEM. HR, heart rate, SBP, systolic blood pressure, DBP, diastolic blood pressure and MAP, mean arterial pressure.

# *P*<0.05 vs Control;

† *P*<0.05 vs OVX+SHAM_ET_;

‡ *P*<0.05 vs OVX+SHAM_SED_;

$ *P*<0.05 vs OVX+MI_SED_.

### Analysis of Oxidative Stress by the Dihydroethidium Fluorescence

**Figure 3:**
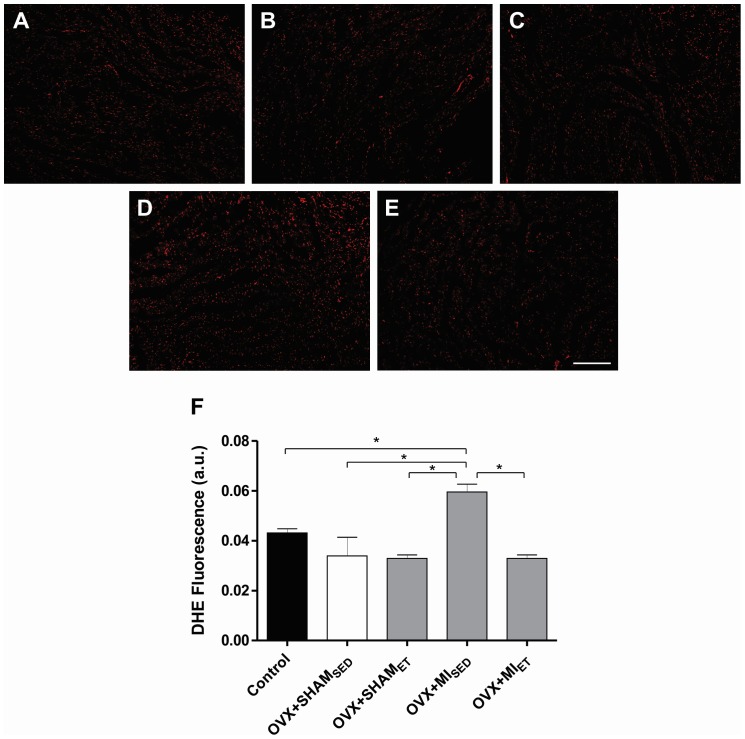
Analysis of oxidative stress in cardiac tissue. Analysis of superoxide formation in sections of cardiac tissue by the dihydroethidium fluorescence. Representative images of Control (A), OVX+SHAM_SED_ (B), OVX+SHAM_ET_ (C), OVX+MI_SED_ (D) and OVX+MI_ET_ (E) groups. Data are expressed as mean ± SEM (n = 4). *P<0.05. Bar: 200 µm.

Analysis of superoxide formation showed a significant increase in the fluorescence of OVX+MI_SED_ when compared with other groups. However, in the OVX+MI_ET_ group, eight weeks of training prevents the increase in oxidative stress promoted by MI (Fig. 3A-F), which demonstrates the efficacy of ET in the maintenance of ROS homeostasis.

### Protein Expression

**Figure 4:**
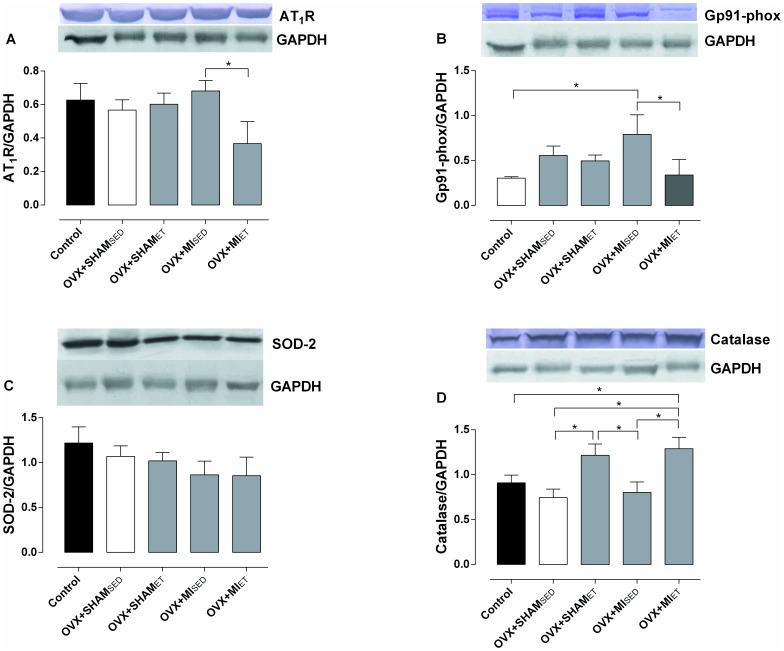
Oxidant and antioxidants proteins expression. Expression of AT1 receptor (A), Gp91phox (B) and the antioxidant enzymes SOD-2 (C) and catalase (D). The densities were normalized to the control protein GAPDH. Data are expressed as mean ± SEM (n = 5). *P<0.05.

The expression of pro-oxidant and antioxidant enzymes was verified in order to analyze the possible mechanisms involved in the preservation of cardiac function. The expression of the AT1 receptor was significantly reduced in the OVX+MI_ET_ group compared with the OVX+MI_SED_ group (Fig. 4A). There was a significant increase in Gp91phox protein expression in MI rats compared to control group. On the other hand, ET was able to significantly reduce the expression of Gp91phox in MI rats (Fig. 4B). No significant differences in SOD-2 expression were found (Fig. 4C). However, a significant increase was observed in catalase expression in the OVX+SHAM_ET_ compared to OVX+MI_SED_ and OVX+SHAM_SED_ and in the OVX+MI_ET_ group compared with Control, OVX+SHAM_SED_ and OVX+MI_SED_ rats (Fig. 4D).

### Evaluation of Interstitial Collagen and Myocyte Hypertrophy

**Figure 5:**
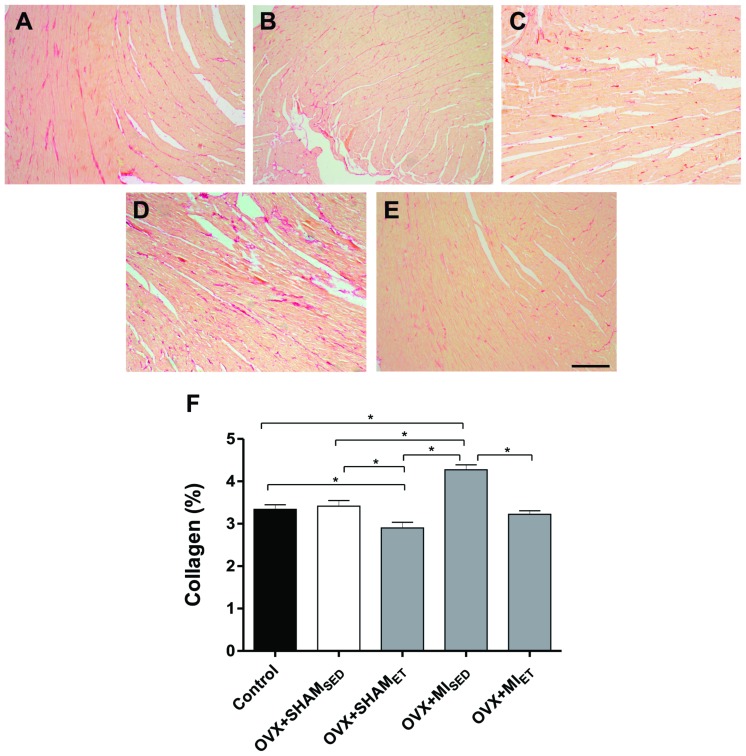
Interstitial collagen deposition evaluation in rat hearts. Representative images of histological sections stained with Picrosirius of Control (A), OVX+SHAM_SED_ (B), OVX+SHAM_ET_ (C), OVX+MI_SED_ (D) and OVX+MI_ET_ (E) groups. Data are expressed as mean ± SEM (n = 7). *P<0.05. Magnifier 400x. Bar: 200 µm.

**Figure 6:**
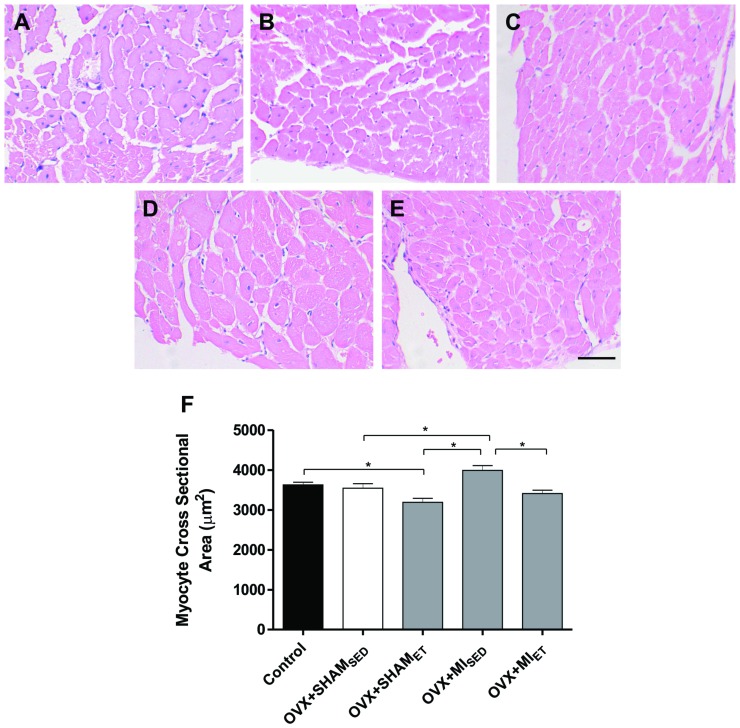
Myocyte cross sectional area evaluation. Representative images of histological sections stained with hematoxylin and eosin of Control (A), OVX+SHAM_SED_ (B), OVX+SHAM_ET_ (C), OVX+MI_SED_ (D) and OVX+MI_ET_ (E) groups. Data are expressed as mean ± SEM (n = 7). *P<0.05. Magnifier 400x. Bar: 50 µm.

The myocardial area occupied by collagen was evaluated in transverse sections of LV stained with Picrosirius. The images were represented in Fig. 5. A significant increase in collagen deposition was observed in MI rats compared with the Control, OVX+SHAM_SED_ and OVX+SHAM_EF_ groups (Fig. 5A, B, C and D). However, exercise training reduced myocardial collagen deposition after MI (Fig. 5E). Fig. 6 shows representative histological sections stained with hematoxylin and eosin. After MI, a significant increase in myocyte cross sectional area was observed when compared to OVX+SHAM_SED_ and OVX+SHAM_ET_ groups (Fig. 6B, C and D). However, ET in MI rats was able to prevent the myocyte hypertrophy (Fig. 6E).

## Discussion

Many studies have investigated ET as a strategy to modify the remodeling process after MI. [14], [16], [17] Therefore, the aim of this study was analyze the role of ET in the prevention or attenuation of cardiac dysfunction and the pathological remodeling process in ovariectomized rats after MI, investigating the possible mechanisms involved in these processes.

The present study has three major findings: i) ET improved the parameters of cardiac function in ovariectomized rats after MI; ii) ET attenuated the effects of MI-induced remodeling; and iii) ET decreased the protein expression of one of the main pathways generating reactive oxygen species and also increased the antioxidant enzyme catalase, which contributes to both improved cardiac function and to the remodeling process.

The enhancement of collagen deposition plays an important role in adverse remodeling after MI. [25] In our study, the animals subjected to eight weeks of ET showed a reduction in collagen deposition compared to the sedentary group. A mechanism that may explain the beneficial effects of ET after MI is the reduction in RAAS activation. The neurohumoral cascade after ischemic cardiac events increases the production of AngII by fibroblasts. [12] The effects of AngII are exerted by the activation of two receptor subtypes (AT1 and AT2) where the effects of AT1 subtype predominate over AT2. [26] Once activated in cardiac cells, the AT1 receptor causes an increase in collagen deposition via multiple signaling pathways. [27]

These effects may be exacerbated in the setting of ovarian hormone deficiency, as is the case in postmenopausal women. [3], [28], [29] Pedram et al., [30] showed that reduction in circulating estrogen levels increases AngII and endothelin-1 production by fibroblasts, macrophages and the endothelium. Their actions mediate transforming growth factor β (TGF-β) which stimulates both matrix metalloproteinase production and the modification of fibroblasts into myofibroblasts, a process that culminates in the synthesis of collagen types I and III. It is noteworthy that the normal adult heart is composed of approximately 2 to 4% collagen, the presence of which confers high tensile strength, and slight changes in the heart's composition may adversely affect cardiac contractility; therefore, the higher the collagen concentration, the worse the contractile force exerted by the myocardium. [24]

A study conducted by Wenhan Wan et al, [31] evaluated how ET attenuates RAAS activation and the subsequent remodeling process after MI. They showed that ET reduces circulating levels of renin and angiotensin converting enzyme (ACE) as well as plasmatic concentrations of AngII and aldosterone, which are associated with the preservation of cardiac function. These effects are independent of the time the training starts (1 or 6 weeks after MI). Similarly, Braith et al., [32] demonstrated that 16 weeks of training decreases circulating levels of AngII in patients with heart failure after MI. It is important to note that although we didn't evaluate the various components of RAAS, the reduction in AT1 receptor expression suggests that ET reduces collagen deposition via this process.

As demonstrated in our study, the increase in collagen deposition in MI animals was accompanied by the reduction of both contraction force (+ dP/dt) as well as an increase in LVEDP, as described by others. [33], [34], [35] In another study, it was demonstrated that an increase in collagen deposition contributes to ventricular chamber strain enhancement and compliance reduction. [25] Therefore, collagen reduction plays a key role in reducing adverse remodeling after MI, [12] and participates in the normal distribution of contraction force during the cardiac cycle. [36]

The activation of the neurohumoral cascade, as previously described, exerts many adverse effects after MI, including cardiac hypertrophy. [37] These effects have been demonstrated by studies showing that the increases in AT1 receptor expression and AngII after MI, as well as ovarian hormone reduction, increase the expression of endothelin receptor type B, resulting in myocardial hypertrophy. [38], [39] Moreover, the overexpression of AT1 receptors in fibroblasts of adult rats induces hypertrophy and remodeling. [37] Estrogen deficiency didn't seems to play an important role in this process, because it was not detected difference in cross sectional area and in the AT1 receptor expression between the ovariectomized and control groups. However, it was been previously reported that the lack of estradiol increases the density of this receptor in rats. [40] Nevertheless, other factors may also contribute to these effects, such as oxidative stress.

Oxidative stress is defined as an imbalance between pro- and antioxidant systems, an imbalance that favors the former and causes cellular damage via an increase in ROS formation. After MI, ROS production is markedly enhanced, as showed by DHE fluorescence. [12] NADPH oxidase is one of the main sources of superoxide production. [41] This complex possesses two membrane bound subunits (Gp91phox and p22phox), as well as more cytosolic subunits which regulate and organize the complex in the membrane, enhancing its activity and producing superoxide. [42]

In the heart, Gp91phox plays a key role in remodeling after MI. [43] It has been previously demonstrated that the activation of the AT1 receptor induces an enhancement in superoxide production by NADPH oxidase, causing hypertrophy by a mechanism dependent on Akt and Rac-1 in conjunction with Gp91phox activation. [41], [42], [44] Moreover, the pro-fibrotic effect triggered by the AT1 receptor has been shown to be mediated by Gp91phox. [45] Observations made by Yung et al., [46] illustrate that ovarian hormone deficiency increases ACE, AT1 receptor, Gp91phox and p22phox expression, as well as plasmatic AngII concentrations, events followed by an enhancement in oxidative stress and a reduction in nitric oxide bioavailability. It is important to note that estrogens are antioxidant compounds, and their reduction contributes to ROS elevation, an additional problematic effect of MI. [47]

In our study, ET decreases Gp91phox expression in OVX+IM_ET_ animals, and this reduction was accompanied by a reduction in collagen deposition and myocyte hypertrophy. A study conducted by Barbosa et al., [18] showed that obese rats subjected to 8 weeks of ET demonstrated reduced superoxide production in parallel with increases in SOD and glutathione peroxidase activity. Similar results were observed by Pinho et al., [48] showing that different training protocols were efficient in decreasing superoxide production in male rats after MI.

Antioxidant enzymes play a key role in ROS homeostasis. [49] We also demonstrated that in addition to Gp91phox reduction, there were improvements in the enzymatic antioxidant system in the setting of ET, as demonstrated by the increase in catalase expression. In a recent study, we demonstrated that eight weeks of swimming training increases the expression of catalase in the coronary arteries of OVX rats. [50] These results suggest that catalase may be more sensitive to ET, contributing to the beneficial effects of ET on the cardiovascular system, although it cannot found difference in the OVX+SHAM_ET_ group. This result, and the lack of improvements in cardiac function and myocyte hypertrophy can be explained by the intensity employed in our protocol, which is considered for Sham animals as a low-intensity exercise and for MI rats as a moderate intensity exercise. Moreover, as the level of oxidative stress during exercise as well as the degree of cardiovascular adaptations in response to exercise depends on the intensity practiced, it would be expected that the highest intensity practiced by MI rats could induce the upregulation in the expression of catalase and the cardiac improvements. Accordingly, the mechanism related to the prevention of oxidative stress in the left ventricle of OVX+MI_ET_ group, demonstrated by DHE fluorescence, seems to occur by the reduction of the pro-oxidant pathway in conjunction with the increase in the expression of antioxidant enzyme catalase.

Therefore, according to the data discussed, we observe that RAAS, and consequently oxidative stress, seem to be the central mechanisms of pathological responses in the cardiovascular system, including MI, and are also associated with the reduction in ovarian hormones. [12], [30] This result may explain why women affected by MI after menopause exhibit a worse prognosis compared to other patients. Therefore, eight weeks of ET seems to attenuate worsening cardiac function in MI rats, even in the setting of ovarian hormone deficiency.

## Conclusions

We concluded that eight weeks of treadmill ET is related to improvements in functional cardiac parameters and to the attenuation of cardiac remodeling in ovariectomized rats after MI. Furthermore, the possible mechanisms involved in these improvements seem to be related to reductions in both AT1 receptor and Gp91phox enzyme expression, as well as an increase in the expression of the antioxidant enzyme catalase, which contributes to a reduction in oxidative stress. Therefore, ET may be an important therapeutic target in the prevention of heart failure in postmenopausal women affected by MI.
