# Correction: Exercise Training Reduces Cardiac Dysfunction and Remodeling in Ovariectomized Rats Submitted to Myocardial Infarction

**DOI:** 10.1371/journal.pone.0118246

**Published:** 2015-02-13

**Authors:** The PLOS ONE Staff

The third author’s name is incorrect; the correct name is Vinícius Mengal. The correct citation is: Almeida SAd, Claudio ERG, Mengal V, Oliveira SGd, Merlo E, Podratz P, et al. (2014) Exercise Training Reduces Cardiac Dysfunction and Remodeling in Ovariectomized Rats Submitted to Myocardial Infarction. PLoS ONE 9(12): e115970. doi:10.1371/journal.pone.0115970
